# Tópicos Emergentes em Insuficiência Cardíaca: Insuficiência Cardíaca com Fração de Ejeção Preservada e Intermediária

**DOI:** 10.36660/abc.20201105

**Published:** 2020-11-01

**Authors:** Miguel Morita Fernandes-Silva, Ricardo Mourilhe-Rocha, Flávio de Souza Brito, Antonio José Lagoeiro Jorge, Victor Sarli Issa, Luiz Cláudio Danzmann

**Affiliations:** 1 Universidade Federal do Paraná CuritibaPR Brasil Universidade Federal do Paraná (UFPR), Curitiba, PR - Brasil; 2 Pontifícia Universidade Católica do Paraná CuritibaPR Brasil Pontifícia Universidade Católica do Paraná (PUCPR), Curitiba, PR - Brasil; 3 Universidade do Estado do Rio de Janeiro Rio de JaneiroRJ Brasil Universidade do Estado do Rio de Janeiro (UERJ), Rio de Janeiro, RJ – Brasil; 4 Hospital Pró-Cardíaco Rio de JaneiroRJ Brasil Hospital Pró-Cardíaco, Rio de Janeiro, RJ - Brasil; 5 Universidade Estadual Paulista Júlio de Mesquita Filho BotucatuSP Brasil Universidade Estadual Paulista Júlio de Mesquita Filho (Unesp), Botucatu, SP - Brasil; 6 INDACOR Centro de Pesquisa Clínica IndaiatubaSP Brasil Centro de Pesquisa Clínica - INDACOR, Indaiatuba, SP - Brasil; 7 Universidade Federal Fluminense NiteróiRJ Brasil Universidade Federal Fluminense (UFF), Niterói, RJ – Brasil; 8 Universidade da Antuérpia Antuérpia Bélgica Universidade da Antuérpia, Antuérpia – Bélgica; 9 Universidade Luterana do Brasil CanoasRS Brasil Universidade Luterana do Brasil (Ulbra), Canoas, RS - Brasil; 10 Hospital São Lucas da PUC-RS Porto AlegreRS Brasil Hospital São Lucas da PUC-RS, Porto Alegre, RS - Brasil

**Keywords:** Insuficiência Cardíaca, Fração de Ejeção Preservada, Fração de Ejeção Intermediária

## Diagnóstico da Insuficiência Cardíaca com Fração de Ejeção Preservada (ICFEP)

As recomendações diagnósticas atuais requerem evidências de congestão ou de baixo débito cardíaco, considerando uma combinação de informações clínicas, eletrocardiograma, exames de imagem, biomarcadores e o teste de exercício hemodinâmico em casos selecionados.^1^

**Figure 1:**
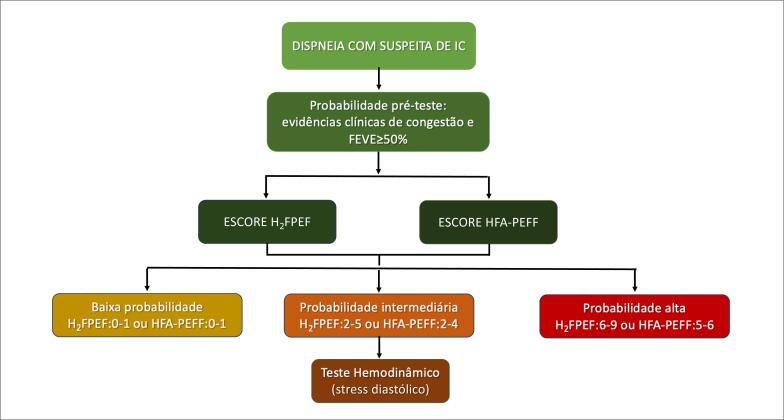
Fluxograma diagnóstico para insuficiência cardíaca com fração de ejeção preservada (ICFEP).

Recomenda-se uma abordagem clínica pré-teste (Etapa 1), seguida de um escore confirmatório (Etapa 2), com a finalidade de confirmar ou descartar o diagnóstico de ICFEP. O teste de exercício hemodinâmico (Etapa 3) é indicado em pacientes com pontuação intermediária^2^ (Figura 1).

## Abordagem Clínica Inicial − Etapa 1

A dispneia e a fadiga demandam anamnese e exame físico minuciosos. Eletrocardiograma, radiografia de tórax, ecocardiograma, peptídeos natriuréticos e teste cardiopulmonar são sugeridos para elaborar probabilidade clínica pré-teste concreta de ICFEP ou para descartá-la.

## Pontuações Confirmatórias − Etapa 2

Dois sistemas de pontuação, o escore H_2_FPEF e o escore HFA-PEFF, foram recentemente desenvolvidos para estabelecer a probabilidade de diagnóstico.

A pontuação H_2_FPEF foi derivada a partir da identificação de variáveis clínicas e de imagem, independentemente associadas com o diagnóstico invasivo de ICFEP em uma coorte populacional (Tabela 1).

**Tabela 1:** Escore H^2^FPEF.

	Variável Clínica	Característica	Pontos
**H2**	Obesidade (*heavy*)	IMC > 30 Kg/m^2^	**2**
	Hipertensão	2 ou mais anti-hipertensivos	**1**
**F**	Fibrilação atrial	Paroxística ou persistente	**3**
**P**	Hipertensão Pulmonar	PSAP > 35 mmHg (ecocardiograma)	**1**
**E**	Idade avançada (*Elderly*)	Idade > 60 anos	**1**
**F**	Pressões de Enchimento (*Filling pressures*)	E/é> 9	**1**

IMC: Índice de massa corpórea; PSAP: pressão sistólica da artéria pulmonar.

O escore HFA-PEFF é composto por índices ecocardiográficos morfológicos, funcionais e biomarcadores séricos. São critérios menores e maiores, que somam 1 ou 2 pontos cada, respectivamente (Tabela 2).

**Tabela 2:** Escore HFA-PEFF.

CRITÉRIOS	MAIOR (2 pontos)	MENOR (1 ponto)
**FUNCIONAL**	é septal < 7 ou é lateral < 10 ou E/é≥ 15 ou Velocidade RT > 2,8 m/s (PSAP > 35 mmHg)	E/é 9-14 ou GLS < 16%
**MORFOLÓGICO**	VAEI > 34 mL/m^2^ ou Massa VE ≥ 149/122 g/m^2^ (H/M) e RWT > 0,42	VAE 29 - 34 mL/m^2^ ou Massa VE > 115/95 g/m^2^ (H/M) ou RWT > 0,42 ou Septo ou PP ≥ 12 mm
**BIOMARCADOR (Ritmo sinusal)**	NT-proBNP > 220 pg/mL ou BNP > 80 pg/mL	NT-proBNP 125 - 220 pg/mL ou BNP 35 - 80 pg/mL
**BIOMARCADOR (Fibrilação atrial)**	NT-proBNP > 660 pg/mL ou BNP > 240 pg/mL	NT-proBNP 365 - 660 pg/mL ou BNP 105 - 240 pg/mL

BNP: peptídeo natriurético do tipo B; NT-proBNP: peptídeo natriurético N-terminal pró-tipo B; VAEi: índice de volume atrial esquerdo; IMVE: índice de massa ventricular esquerda; H: homens / M: mulheres; GLS: ***strain global*** longitudinal; velocidade RT: velocidade do fluxo de regurgitação da valva tricúspide.

Nessa estratégia, ICFEP pode ser praticamente afastada em pacientes com escores baixos (0 ou 1). Por outro lado, o diagnóstico de ICFEP pode ser estabelecido em pacientes com escores elevados (H_2_FPEF ≥ 6 ou HFA-PEFF ≥ 5).^3^ Nos pacientes com pontuações intermediárias (H_2_FPEF 2 a 5 ou HFA-PEFF 2 a 4) pode ser necessária a realização do teste de exercício hemodinâmico^4^ (Figura 1).

## Teste Hemodinâmico de Exercício − Etapa 3

Nesta etapa, o paciente é submetido a um teste de esforço diastólico inicialmente não invasivo. Os índices selecionados são: E/e’ − estimativa da pressão de enchimento do ventrículo esquerdo (VE) e velocidade de regurgitação da valva tricúspide (VRT); e estimativa não invasiva da pressão sistólica da artéria pulmonar. Ao atingir o ponto de corte, acrescenta-se pontuação adicional à pontuação obtida na Etapa 2 (2 pontos se E/e’ ≥ 15; 3 pontos se E/e’ ≥ 15 e VRT > 3,4 m/s). Se a soma for 5, ele atende ao critério de diagnóstico. Em casos selecionados, o teste de estresse diastólico invasivo também pode ser realizado.^4^

## Etiologia da ICFEP

Ao rotularmos todos os pacientes com sintomas de insuficiência cardíaca (IC) e FEVE ≥ 50% como ICFEP, estamos admitindo que existe um denominador fisiopatológico comum entre eles, o que não é uma verdade. Esses pacientes apresentam fisiopatologia complexa, que inclui aumento da resistência vascular sistêmica e da rigidez arterial, acoplamento ventricular-arterial anormal, redução da função sistólica no longo eixo do VE, diminuição do relaxamento ventricular, redução da complacência do VE, alteração da função contrátil do ventrículo direito (VD) e incompetência cronotrópica.^4^

A ICFEP também apresenta uma grande heterogeneidade fenotípica, que envolve uma combinação de fatores de risco e comorbidades, que pode influenciar o prognóstico e o tratamento.^5^

Podemos dividir a etiologia da ICFEP em forma primária, causada por fatores metabólicos e hemodinâmicos comuns com estratégias terapêuticas mais uniformes, e em forma secundária, envolvendo os casos menos comuns com uma etiologia específica, como as cardiomiopatias hereditárias, infiltrativas, restritivas, inflamatórias ou genéticas e que tenha os critérios diagnósticos de ICFEP (Tabela 3).^4^^,^^6^

**Tabela 3:** Etiologias da insuficiência cardíaca com fração de ejeção preservada

Etiologias	Características	Causas
**ICFEP primária**	Mulheres e idosos com fatores metabólicos e hemodinâmicos comuns.	Hipertensão arterial, diabetes, obesidade.
**ICFEP secundária**	Etiologia específica	
**Cardiomiopatias infiltrativas**	Relacionadas ou não à malignidade.	Metástases, doença de Fabry, doença de Danon, doença de Pompe.
**Cardiomiopatias restritivas**		Amiloidose, sarcoidose, radiação, esclerodermia.
**Cardiomiopatias inflamatórias e autoimunes**	Relacionadas ou não à infecção.	Vírus cardiotrópicos, doenças autoimunes, miocardite linfocítica.
**Cardiomiopatias hereditárias e genéticas**		Cardiomiopatia hipertrófica, distrofia muscular (Duchenne).
**Isquêmicas**		Disfunção endotelial e microvascular, pós-infarto do miocárdio.
**Tóxica**	Abuso de substâncias, metais pesados, medicamentos.	Álcool, cocaína, ferro, cloroquina, antraciclinas.
**Outras**	Estados de alto débito, sobrecarga de volume, desordens do ritmo cardíaco.	Tireotoxicose, fistula arteriovenosa, arritmias ventriculares e atriais, anemia severa, doença de Paget.

ICFEP: insuficiência cardíaca com fração de ejeção preservada.

## Recomendações para o Tratamento da IC com Fração de Ejeção Intermediária (ICFEI)

Os ensaios clínicos randomizados (ECR) em ICFEP avaliaram o uso de IECA, BRA e antagonista mineralocorticoide e não mostraram superioridade ao placebo na redução de desfechos relacionados à IC.^1^^,^^7^^,^^8^ ^11^^,^^12^ De maneira semelhante, o sacubitril-valsartan não foi superior ao valsartan na redução do desfecho composto de hospitalizações por IC ou por morte cardiovascular.^13^^–^^15^

No entanto, análises *post-hoc* desses ECRs demonstraram que terapias correntemente indicadas para o tratamento da ICFER (FEVE < 40%) podem ser extrapoladas a pacientes com ICFEI (FEVE 40-49%).

Nesse sentido, a subanálise do TOPCAT sugeriu benefício da espironolactona em pacientes com FEVE entre 44-50%;^7^ e a subanálise do CHARM revelou benefício com candesartan nos pacientes com FEVE de 40% a 49%.^8^ Em uma metanálise de 11 ECRs, os betabloqueadores estiveram associados à menor mortalidade em pacientes com ICFEI e ritmo sinusal.^9^ Recentemente, uma análise combinada do PARAGON-HF e PARADIGM-HF sugeriu que o sacubitril-valsartana associou-se à redução do desfecho primário em níveis intermediários de FEVE, sendo esse efeito observado em níveis mais elevados de FEVE em mulheres do que em homens. Esses dados sugerem que o sacubitril-valsartana possa ser benéfico para pacientes com ICFEI, especialmente do gênero feminino.^10^

## Perspectivas para o Tratamento da ICFEP

As mesmas subanálises dos ECRs citadas acima indicaram, de forma consistente, a ausência de benefício dessas medicações em pacientes com IC e FEVE mais elevada (> = 50%).^8^^–^^10^^,^^16^ É possível que a ausência de benefício resulte da heterogeneidade de fenótipos, da presença de múltiplas comorbidades e da diversidade de mecanismos envolvidos na progressão da doença. Assim, o tratamento das comorbidades, como isquemia miocárdica, fibrilação atrial e hipertensão, é fundamental para aliviar sintomas e potencialmente reduzir a progressão da ICFEP.^16^

Atualmente, os ECRs avaliam os desfechos de dois inibidores da SGLT2 – dapagliflozina e empagliflozina – e dois antagonistas mineralocorticoides – espironolactona e finerenona – em pacientes com ICFEP.^17^

## Lista de Participantes do Heart Failure Summit Brazil 2020 / Departamento de Insuficiência Cardíaca - DEIC/SBC

Aguinaldo Freitas Junior, Andréia Biolo, Antonio Carlos Pereira Barretto, Antônio Lagoeiro Jorge, Bruno Biselli, Carlos Eduardo Montenegro, Denilson Campos de Albuquerque, Dirceu Rodrigues de Almeida, Edimar Alcides Bocchi, Edval Gomes dos Santos Júnior, Estêvão Lanna Figueiredo, Evandro Tinoco Mesquita, Fabiana G. Marcondes-Braga, Fábio Fernandes, Fabio Serra Silveira, Felix José Alvarez Ramires, Fernando Atik, Fernando Bacal, Flávio de Souza Brito, Germano Emilio Conceição Souza, Gustavo Calado de Aguiar Ribeiro, Humberto Villacorta Jr., Jefferson Luis Vieira, João David de Souza Neto, João Manoel Rossi Neto, José Albuquerque de Figueiredo Neto, Lídia Ana Zytynski Moura, Livia Adams Goldraich, Luís Beck-da-Silva, Luís Eduardo Paim Rohde, Luiz Claudio Danzmann, Manoel Fernandes Canesin, Marcelo Bittencourt, Marcelo Westerlund Montera, Marcely Gimenes Bonatto, Marcus Vinicius Simões, Maria da Consolação Vieira Moreira, Miguel Morita Fernandes da Silva, Monica Samuel Avila, Mucio Tavares de Oliveira Junior, Nadine Clausell, Odilson Marcos Silvestre, Otavio Rizzi Coelho Filho, Pedro Vellosa Schwartzmann, Reinaldo Bulgarelli Bestetti, Ricardo Mourilhe Rocha, Sabrina Bernadez Pereira, Salvador Rassi, Sandrigo Mangini, Silvia Marinho Martins, Silvia Moreira Ayub Ferreira, Victor Sarli Issa.
