# Supplementary material for: Tópicos Emergentes em Insuficiência Cardíaca: Insuficiência Cardíaca com Fração de Ejeção Preservada e Intermediária
Source: Arq Bras Cardiol. 2020 Nov 1;115(5):949–52. [Article in Portuguese] doi: 10.36660/abc.20201105 (PMC8452211; doi:10.36660/abc.20201105)
Supplement: Translation

# Translation: Emerging Topics in Heart Failure: Heart Failure With Preserved and Mid-Range Ejection Fraction

**Authors:** Miguel Morita Fernandes-Silva, Ricardo Mourilhe-Rocha, Flávio de Souza Brito, Antonio José Lagoeiro Jorge, Victor Sarli Issa, Luiz Cláudio Danzmann

**Affiliations:** 1 CuritibaPR Brazil Universidade Federal do Paraná (UFPR), Curitiba, PR - Brazil; 2 CuritibaPR Brazil Pontifícia Universidade Católica do Paraná (PUCPR), Curitiba, PR - Brazil; 3 Rio de JaneiroRJ Brazil Universidade do Estado do Rio de Janeiro (UERJ), Rio de Janeiro, RJ – Brazil; 4 Rio de JaneiroRJ Brazil Hospital Pró-Cardíaco, Rio de Janeiro, RJ - Brazil; 5 BotucatuSP Brazil Universidade Estadual Paulista Júlio de Mesquita Filho (Unesp), Botucatu, SP - Brazil; 6 IndaiatubaSP Brazil Centro de Pesquisa Clínica - INDACOR, Indaiatuba, SP - Brazil; 7 NiteróiRJ Brazil Universidade Federal Fluminense (UFF), Niterói, RJ – Brazil; 8 Antuérpia Bélgica Universidade da Antuérpia, Antuérpia – Bélgica; 9 CanoasRS Brazil Universidade Luterana do Brasil (Ulbra), Canoas, RS - Brazil; 10 Porto AlegreRS Brazil Hospital São Lucas da PUC-RS, Porto Alegre, RS - Brazil

**Keywords:** Heart Failure, Preserved Ejection Fraction, Mid-Range Ejection Fraction

## Heart Failure with Preserved Ejection Fraction (HFpEF) Diagnosis

Current diagnostic recommendations require evidence of congestion or low cardiac output, considering a combination of clinical information, electrocardiogram, imaging, biomarkers, and, in selected cases, hemodynamic exercise testing.^1^

A pretest clinical approach (step 1) followed by a confirmatory score (step 2) is recommended to confirm or rule out the diagnosis of HFpEF. Hemodynamic exercise testing (step 3) is indicated for patients with an intermediate score^2^ (Figure 1).

**Figure 1:** Diagnostic algorithm for HFpEF.

## Pretest Clinical Approach – step 1

Evaluation of dyspnea and fatigue requires a detailed history and physical examination. Electrocardiogram, chest radiography, echocardiogram, natriuretic peptides, and cardiopulmonary testing are suggested to define the clinical pretest probability of HFpEF or rule it out altogether.

## Confirmatory Scores – step 2

Two scoring systems, the H2FPEF score and the HFA-PEFF score, have recently been developed to establish the probability of HFpEF diagnosis.

The H2FPEF score was derived from selected clinical and imaging variables independently associated with the invasive diagnosis of HFpEF in a population-based cohort (Table 1).

**Table 1:** H^2^FPEF score.

Score Label	Clinical Variable	Characteristics	Points
**H2**	Heavy	BMI > 30 Kg/m^2^	**2**
	Hypertension	2 or more anti-hypertensive drugs	**1**
**F**	Atrial fibrillation	Paroxistic or Persistent	**3**
**P**	Pulmonary hypertension	PASP > 35 mmHg (measured on Doppler echo)	**1**
**E**	Elderly	Age > 60 years	**1**
**F**	Filling pressures	E/é> 9 (measured on Doppler echo)	**1**

BMI: body mass index; PASP: pulmonary artery systolic pressure.

The HFA PEFF score is composed of morphological and functional echocardiographic measures and serum biomarker criteria. There are minor and major criteria, which add up to 1 or 2 points each, respectively (Table 2).

**Table 2:** HFA-PEFF score

DOMAIN	MAJOR CRITERIA (2 poInts)	MINOR CRITERIA (1 poInt)
**FUNCTIONAL**	é septal < 7 or é lateral < 10 or E/é≥ 15 or RT velocity > 2,8 m/s (PSAP > 35 mmHg)	E/é 9-14 or GLS < 16%
**MORPHOLOGIC**	LA Vol index > 34 mL/m^2^ or LVMI ≥ 149/122 g/m^2^ (H/M) and RWT > 0,42	LA Vol index 29 - 34 mL/m^2^ ou LVMI > 115/95 g/m^2^ (H/M) or RWT > 0,42 or left ventricle wall thinckness ≥ 12 mm
**BIOMARKER (sinusal rythm)**	NT-proBNP > 220 pg/mL or BNP > 80 pg/mL	NT-proBNP 125 - 220 pg/mL or BNP 35 - 80 pg/mL
**BIOMARKER (atrial fibrillations)**	NT-proBNP > 660 pg/mL or BNP > 240 pg/mL	NT-proBNP 365 - 660 pg/mL or BNP 105 - 240 pg/mL

BNP: B-type natriuretic peptide; NT-proBNP: N-terminal pro–B-type natriuretic peptide; LA vol: left atrial volume; LVMI: left ventricular mass index; RWT: relative wall thickness; M: men / W: women; GLS: global longitudinal strain; RT: velocity of tricuspid valve regurgitation flow.

In this strategy, HFpEF can be ruled out in patients with low scores (0 or 1). Conversely, the diagnosis of HFpEF can be established in patients with high scores (H2FPEF ≥ 6 or HFA PEFF ≥ 5).^3^ In patients with intermediate scores (H2FPEF 2 to 5 or HFA PEFF 2 to 4), a hemodynamic exercise test may be necessary^4^ (Figure 1).

## Hemodynamic Exercise Testing – Step 3

At this stage, the patient undergoes an initially non-invasive diastolic stress test. The selected indexes are E/e’, which estimates the LV filling pressure, and the tricuspid valve regurgitation speed (VRT), which estimates the pulmonary artery systolic pressure. Upon reaching the cutoff point, an additional score is added to that obtained in step 2 (2 points if E/e’ ≥15; 3 points if E/e’ ≥15 and VRT >3.4 m/s). If the final sum is 5 or above, the patient meets diagnostic criteria for HFpEF. In selected cases, an invasive diastolic stress test can also be performed.^4^

## Etiology of HFpEF

By labeling all patients with symptoms of HF and LVEF ≥50% as having HFpEF, we are assuming a common pathophysiological denominator among these patients, which is not true. Patients with HFpEF display a complex pathophysiology which includes increased systemic vascular resistance, increased arterial stiffness, abnormal ventricular-arterial coupling, reduced systolic function in the long axis of the LV, decreased ventricular relaxation, reduced LV compliance, abnormal RV contractile function, and chronotropic incompetence.^4^

HFpEF has wide phenotypic heterogeneity, with a combination of risk factors and comorbidities that may affect prognosis and treatment.^5^

The etiology of HFpEF can be divided into a primary form, which shares common metabolic and hemodynamic characteristics and similar therapeutic strategies, and another form that may be called secondary, which is less common and has specific etiologies, such as hereditary, infiltrative, restrictive, inflammatory or genetic cardiomyopathies.^4^^,^^6^ (Table 3).

**Table 3:** Etiologies of heart failure with preserved ejection fraction

Etiologies	Characteristic	Causes
**Primary HFpEF**	Female sex, older age Common metabolic and hemodynamic factors	Hypertension, diabetes, obesity
**Secondary HFpEF**	Specific etiology	
**Infiltrative cardiomyopathies**	Related or not to malignancy	Metastasis, Fabry disease, Danon disease, Pompe disease
**Restrictive cardiomyopathies**		Amyloidosis, sarcoidosis, radiation, scleroderma
**Inflammatory and autoimmune cardiomyopathies**	Related or not to infection	Cardiotropic viruses, autoimmune diseases, lymphocytic myocarditis
**Hereditary and Genetic Cardiomyopathies**		Hypertrophic cardiomyopathy, Duchenne muscular dystrophy
**Ischemic disease**		Endothelial and microvascular dysfunction after myocardial infarction
**Toxic**	Substance abuse; heavy metals; medicines	Alcohol, cocaine, iron, chloroquine, anthracyclines
**Others**	High-output state; volume overload; heart rhythm disorders	Thyrotoxicosis, arteriovenous fistula, ventricular and atrial arrhythmias, severe anemia, Paget's disease

HFpEF: heart failure with preserved ejection fraction.

## Recommendations for the Treatment of HFmrEF

Randomized clinical trials (RCT) in HFpEF evaluated the use of ACEI, ARB, and mineralocorticoid antagonists; none proved superior to placebo in reducing HF-related adverse outcomes.^1^^,^^7^^,^^8^^,^^11^^,^^12^ Similarly, sacubitril-valsartan was not superior to valsartan alone in reducing the composite outcome of hospitalizations for HF or cardiovascular death.^13^^–^^15^

However, post-hoc analysis from these RCTs suggested that therapies currently indicated for the treatment of HF and reduced ejection fraction (LVEF <40%) can be extrapolated to patients with HF and mid-range ejection fraction (HFmrEF, LVEF 40-49%).

In this sense, a TOPCAT sub-analysis suggested a benefit of spironolactone in patients with LVEF from 44 to 50%,^7^ and a CHARM sub-analysis revealed a benefit with candesartan in patients with LVEF from 40% to 49%.^8^ In a meta-analysis of 11 RCTs, beta-blockers were associated with lower mortality in patients with HFmrEF and sinus rhythm.^9^ Recently, a combined analysis of PARAGON-HF and PARADIGM-HF suggested that sacubitril-valsartan was associated with a reduction in the primary outcome at intermediate (mid-range) levels of LVEF, with this effect seen at higher levels of LVEF in women than in men. These data suggest that sacubitril-valsartan may be beneficial for patients with HFmrEF, especially in women.^10^

## Perspectives in Treatment of HFpEF

The same sub-analysis of the RCTs above consistently indicated no benefit from these medications in patients with HF and higher LVEF (≥ 50%), which is the actual cutoff point for definition of HFpEF in the guidelines.^8^^–^^10^^,^^16^ It is possible that the lack of benefit results from the heterogeneity of phenotypes, the presence of multiple comorbidities, and the diversity of mechanisms underlying disease progression. In this sense, the treatment of comorbidities such as myocardial ischemia, atrial fibrillation, and hypertension is essential to relieving symptoms and potentially reducing the progression of HFpEF.^16^

RCTs to evaluate the effect of two SGLT2 inhibitors (dapagliflozin and empagliflozin) and two mineralocorticoid antagonists (spironolactone and finerenone) on outcomes in patients with HFpEF are ongoing.^17^

## List of participants of the Heart Failure Summit Brazil 2020 / Heart Failure Department - Brazilian Society of Cardiology

Aguinaldo Freitas Junior, Andréia Biolo, Antonio Carlos Pereira Barretto, Antônio Lagoeiro Jorge, Bruno Biselli, Carlos Eduardo Montenegro, Denilson Campos de Albuquerque, Dirceu Rodrigues de Almeida, Edimar Alcides Bocchi, Edval Gomes dos Santos Júnior, Estêvão Lanna Figueiredo, Evandro Tinoco Mesquita, Fabiana G. Marcondes-Braga, Fábio Fernandes, Fabio Serra Silveira, Felix José Alvarez Ramires, Fernando Atik, Fernando Bacal, Flávio de Souza Brito, Germano Emilio Conceição Souza, Gustavo Calado de Aguiar Ribeiro, Humberto Villacorta Jr., Jefferson Luis Vieira, João David de Souza Neto, João Manoel Rossi Neto, José Albuquerque de Figueiredo Neto, Lídia Ana Zytynski Moura, Livia Adams Goldraich, Luís Beck-da-Silva Neto, Luís Eduardo Paim Rohde, Luiz Claudio Danzmann, Manoel Fernandes Canesin, Marcelo Bittencourt, Marcelo Westerlund Montera, Marcely Gimenes Bonatto, Marcus Vinicius Simões, Maria da Consolação Vieira Moreira, Miguel Morita Fernandes da Silva, Monica Samuel Avila, Mucio Tavares de Oliveira Junior, Nadine Clausell, Odilson Marcos Silvestre, Otavio Rizzi Coelho Filho, Pedro Vellosa Schwartzmann, Reinaldo Bulgarelli Bestetti, Ricardo Mourilhe Rocha, Sabrina Bernadez Pereira, Salvador Rassi, Sandrigo Mangini, Silvia Marinho Martins, Silvia Moreira Ayub Ferreira, Victor Sarli Issa.
